# Cortical swallowing processing in early subacute stroke

**DOI:** 10.1186/1471-2377-11-34

**Published:** 2011-03-11

**Authors:** Inga K Teismann, Sonja Suntrup, Tobias Warnecke, Olaf Steinsträter, Maren Fischer, Agnes Flöel, E Bernd Ringelstein, Christo Pantev, Rainer Dziewas

**Affiliations:** 1 Department of Neurology, University of Muenster, Albert-Schweitzer-Str.33, 48149 Muenster, Germany; 2 Institute for Biomagnetism and Biosignalanalysis, University of Muenster, Malmedyweg 15, 48149 Muenster, Germany; 3 Department of Neurology, Charite Universitätsmedizin Berlin, Charitéplatz 1, 10117 Berlin, Germany

## Abstract

**Background:**

Dysphagia is a major complication in hemispheric as well as brainstem stroke patients causing aspiration pneumonia and increased mortality. Little is known about the recovery from dysphagia after stroke. The aim of the present study was to determine the different patterns of cortical swallowing processing in patients with hemispheric and brainstem stroke with and without dysphagia in the early subacute phase.

**Methods:**

We measured brain activity by mean of whole-head MEG in 37 patients with different stroke localisation 8.2 +/- 4.8 days after stroke to study changes in cortical activation during self-paced swallowing. An age matched group of healthy subjects served as controls. Data were analyzed by means of synthetic aperture magnetometry and group analyses were performed using a permutation test.

**Results:**

Our results demonstrate strong bilateral reduction of cortical swallowing activation in dysphagic patients with hemispheric stroke. In hemispheric stroke without dysphagia, bilateral activation was found. In the small group of patients with brainstem stroke we observed a reduction of cortical activation and a right hemispheric lateralization.

**Conclusion:**

Bulbar central pattern generators coordinate the pharyngeal swallowing phase. The observed right hemispheric lateralization in brainstem stroke can therefore be interpreted as acute cortical compensation of subcortically caused dysphagia. The reduction of activation in brainstem stroke patients and dysphagic patients with cortical stroke could be explained in terms of diaschisis.

## Background

Swallowing is a complex function involving 5 cranial nerves and 50 different smooth and skeletal muscles. In the neural processing of swallowing both brainstem and cortical areas are involved. Dysphagia is an important complication after ischemic stroke, found in up to 50% of patients with cortical stroke and 67% of patients with a stroke confined to the [1-4]. While many patients experience some form of swallowing recovery within the first few weeks after stroke, 40% of dysphagic stroke patients still develop aspiration pneumonia that increases the use of artificial feeding, the length of hospital stay, and mortality [5]. About 6% of all patients with stroke die from aspiration pneumonia within the first year [6].

In healthy humans several brain imaging studies have examined cortical activation during swallowing using functional magnetic resonance imaging (fMRI), transcranial magnetic stimulation (TMS) and magnetoencephalography (MEG) [7-9]. Consistently across studies, an involvement of the bilateral primary and secondary sensorimotor cortices was found. Additionally, activation of the insular cortex, the frontal operculum, the arterior cingulum, the posterior parietal cortex, and the supplementary motor cortex was observed in different studies [7,8,10-13]. Several studies suggest a task sharing for different components of deglutition between the two hemispheres. The left hemisphere more selectively mediates the oral phase and therefore volitional components, whereas the right hemisphere contributes more to the pharyngeal phase and automatic reflexive aspects of swallowing. First insights into this topic were generated by lesion studies [14] and could be supported by a previous study on healthy subjects by our group [15]. So far, only few studies have examined the cortical swallowing processing in dysphagic patients. Previous studies of our group revealed compensational mechanisms on swallowing in the aging brain [16]. In different acute and also slowly progressive diseases cortical compensational effects could be shown for human deglutition [17,18]. However, the impact of stroke localization on cortical swallowing function has rarely been explored by functional imaging studies. The few existing works on cortical swallowing processing in stroke included only patients with unilateral supratentorial infarct and applied either TMS [19,20] or fMRI [21]. Those studies have been performed at different time points after stroke. Traditionally, acute, subacute, and chronic phases after stroke are distinguished. The time ranges characterizing these three phases strongly vary in literature. Mostly, the first three to seven days are referred to as the acute phase. The first one to six months are defined as the subacute phase, and the chronic phase begins after three or six months in most studies [22-26]. In the present study, subjects were measured 8.2 +/- 4.8 days after stroke. We decided to label this time range "early subacute phase". Alternatively the naming "late acute phase" would be possible.

The aim of the present study was to examine cortical swallowing processing in patients in this early subacute phase after stroke. We hypothesized a decrease of cortical activation in the sensorimotor areas in dysphagic hemispheric stroke patients mainly in the affected hemisphere, due to a disturbance of the swallowing network. In non dysphagic stroke patients, we expected an increase of activation in the contralesional hemisphere, reflecting compensational mechanisms in these sensorimotor areas. According to the hypothesis of hemispheric "task-sharing" in swallowing processing, we expect an increase of cortical activation in the right hemisphere in patients with brainstem stroke as a compensational effect of the disturbance of the central swallowing generators.

## Methods

### Participants

19 patients with mild (penetration of soft solid food) to moderate (penetration of liquids and puree consistency) stroke induced dysphagia were included in the study. Patients showing aspiration of small sips of water during fiberoptic swallowing examination were excluded to avoid aspiration during MEG recordings. 18 stroke patients with no indication for a swallowing disorder were included in the groups of non-dysphagic patients. All 37 patients (25 men, 12 women, age 33-81 years, mean 63.5 +/- 11.8 years) suffered from ischaemic unilateral hemispheric or brainstem stroke, as determined by both magnetic resonance imaging (MRI) and a positive (diffusion weight imaging (DWI) scan, were recruited over the course of 18 months (see table 1). Patients with severe aphasia had to be excluded, because they were not able to give informed consent. We could also not include patients in need of continuous monitoring, or those too immobile to be transported to the MEG laboratory. All patients satisfied the inclusion criteria of (a) being able to give informed consent, (b) having no history of swallowing problems prior to stroke, (c) having no history of neurological disease including previous stroke, (d) taking no neuromuscular modulating drugs, (e) having no other serious intercurrent illness, and (f) having no apparent aspiration in fiberoptic swallowing examination. Handedness before stroke was determined. Neurologic stroke symptoms were assessed at the time point of the MEG measurement using the NIH-SS [27]). Oropharyngeal function was examined clinically by a speech and language therapist including a FEES within 24 hours before MEG measurement. Stroke patients were allocated to different groups depending on stroke localization and ability to swallow. This resulted in 6 patient groups (right hemispheric stroke, left hemispheric stroke, and brainstem stroke with and without dysphagia respectively). MEG measurement was performed on average 8.2 +/- 4.8 days after stroke.

**Table 1:** MRI tomographic findings and clinical details of all 37 patients.

Patient	Age	Sex/Hand	days after stroke	NIH-SS	FEES Score	Location of infarct
**Right hemispheric stroke with dysphagia (RHS +)**						
1	71	F/R	9	13	4	R, fronto-parietal
2	81	F/R	4	6	4	R, multiple infarctions MCA area
3	69	F/R	6	14	4	R, corona radiata, basal ganglia
4	77	F/R	7	10	3	R, thalamus, hippocampus, insula, parietal
5	77	M/R	19	6	4	R, fronto-parietal
6	62	M/L	3	3	3	R, precentral
	72.8 +/- 6.9	2 male	8 +/- 5.8	8.7+/- 4.4	3.5+/-.8	

**Right hemispheric stroke without dysphagia (RHS -)**						
1	46	M/B	9	8	1	R, capsula interna
2	72	M/R	6	3	1	R, parieto-occipital
3	66	M/R	5	3	1	R, corona radiate
4	65	F/R	6	14	1	R, basal ganglia and capsula interna
5	66	M/R	5	5	1	R, postcentral
6	69	M/R	3	5	1	R, parieto-occipal, lat. temporal lobe
7	33	M/R	17	10	1	R, basal ganglia, putamen
	59.6 +/- 14.4	6 male	7.3 +/- 4.6	6.9 +/- 4.1	1(*)	

**Left hemispheric stroke with dysphagia (LHS +)**						
1	70	M/R	11	13	4	L, basal ganglia
2	78	F/R	10	14	4	L, operculum, insula
3	71	M/R	26	11	3	L, precentral
4	71	F/R	14	5	4	L, temporo-parietal
5	69	M/R	6	4	2	L, gyri temporalis sup. and med.
6	48	M/R	6	2	3	L, parieto-occipall incl Insula
7	65	M/R	8	6	2	L, thalamus
8	43	F/R	7	8	2	L, temporo-parietal
	63.6 +/- 13	5 male	6.8 +/- 2.5	7.9 +/- 4.4	3+/-.9	

**Left hemispheric stroke without dysphagia (LHS -)**						
1	79	M/R	8	4	1	L, parieto-occipital
2	60	M/R	4	2	1	L, Ncl. caudatus, insula, precentral
3	53	M/R	7	2	1	L, capsula interna
4	71	F/R	10	4	1	L, precentral
5	67	F/R	5	2	1	L, dorsal hippocampus
6	55	F/R	3	2	1	L, pre- and postcentral
7	57	F/R	7	7	1	L, temporo-parietal
8	51	M/R	4	6	1	L, multiple infarctions MCA area
	61.6 +/- 9.8	4 male	6 +/- 2.4	3.6 +/- 2(*)	1(*)	

**Brainstem stroke with dysphagia (BSS +)**						
1	65	M/R	11	3	2	BS, cerebellum, medulla oblongata
2	69	M/R	8	4	2	BS, left cerebellum, left lat. medulla oblongata
3	65	M/R	9	8	4	BS, left pons, paramedian
4	52	M/R	6	4	3	BS, right mesencephalon
5	76	M/R	8	4	3	BS, right pons
	65.4 +/- 8.7	5 male	8.4 +/- 1.8	4.6 +/- 2	2.8+/-.8	

**Brainstem stroke without dysphagia (BSS -)**						
1	39	M/R	6	4	1	BS, right pons
2	52	M/R	4	3	1	BS, right pons
3	71	M/R	6	3	1	BS, left pons, paramedian
	56.8 +/- 14.2	3 male	5.4 +/- 1.2 (*)	3.3 +/- .6	1(*)	

(*) significant difference from the non dysphagic group with similar stroke location

Seven healthy control subjects (5 male, 2 female, age 66.3 +/- 8.2) without any history of stroke, dysphagia, or any other neurological or ear-nose-throat disorder served as controls. The local ethics committee approved the protocol of the study. Informed consent was obtained from each subject after the nature of the study was explained, in accordance to the principles of the Declaration of Helsinki.

### Fiberoptic endoscopic evaluation of swallowing (FEES)

The basic FEES protocol, as published by Langmore [28] and previously applied in our department [29] was done at bedside with the patient sitting upright within 24 h before MEG measurement. The examination was performed with an Olympus ENF-P4 laryngoscope attached to a camera and a color monitor. All examinations were videotaped. A neurologist experienced in using FEES and a speech therapist jointly completed all FEES procedures. FEES allows visualization of the entire pharyngeal phase of swallowing, except for a very brief period when the contracting pharyngeal walls obstruct the optical tip of the endoscope. Penetration, aspiration, leakage and residues as signs for dysphagia can be evaluated [28]. The endoscopic scoring system introduced by our group was used to evaluate the severity of dysphagia [30]. Briefly, the secretion status is evaluated during endoscopic examination. After that, the patient is successively given standard volumes of puree consistency, liquids, and soft solid food. Based on these observations, stroke-related dysphagia is rated on a 6-point scale according to the risk of penetration or aspiration of the different food consistencies tested. A score of 1 is given if the patient can swallow all three consistencies without problems and equates no dysphagia. Score 2 is given if swallowing of solid food results in penetration or pooling. Score 3 equates that swallowing of liquids results in penetration with sufficient protective reflex. Patients with score 4 show no sufficient protective reflex in swallowing bigger amounts of water. Score 5 is given if swallowing of puree consistency results in penetration and aspiration without protective reflexes. In patients with score 6, swallowing of saliva is not possible without aspiration.

### MEG recording

MEG records grey matter neuronal activity. Cortical neurons transfer information by changes in ionic concentration of electrical currents. If enough parallel neurons are active, the magnetic field of the dipole can be measured from the head surface by MEG. Additionally, MEG demonstrates a high temporal and spatial resolution [31].

A new approach for reconstruction of spatio-temporal brain activities from neuromagnetic measurements is the minimum variance beamformer, which was originally developed in the field of array signal processing, in particular radar and sonar applications [32]. As the MEG beamformer does not rely on averaging (across trials) to increase the signal-to-noise ratio, this method is capable of analyzing both evoked and induced brain activity [33]. Because of its capability to map induced activity, the method is particularly suitable for studying higher cognitive and motor functions where time- and phase-locked brain responses to the stimulus cannot be expected. Another advantage is that experimental paradigms typically set up for fMRI and PET studies can also be used in MEG experiments. An additional advantage of MEG is that subjects can be studied in a sitting position thereby avoiding any potential confounds caused by an experimental design requiring an unphysiological supine position during swallowing. Problematic are the artifacts caused by oropharyngeal muscle activation during deglutition, which make it difficult to study activation in subcortical and bulbar structures [12,34]. However, cortical areas and especially sensorimotor areas can be examined in detail.

In this study, we focused on Synthetic Aperture Magnetometry (SAM), a minimum-variance beamformer with an integrated step for the estimation of source orientation [35], which has been successfully applied in a series of MEG studies during the last few years. Applying SAM volumetric maps of stimulus related brain activity are determined by contrasting active and control states. As a consequence the resulting 3 D images contain functional, but little anatomical information, and therefore co-registration with structural magnetic resonance images (MRI) is necessary to gain the structural information. In the last few years SAM has been demonstrated to be a reliable method to examine complex sensorimotor functions [36], especially swallowing [7,10,12,18], whereas motor tasks result in ERD of the beta-rhythm in cortical motor areas [37].

To facilitate swallowing during MEG measurements water was infused into the mouth of the subjects via a plastic tube of 4.7 mm in diameter, which was attached to a water bag. The tip of the tube was placed in the corner of the mouth between the buccal side of the teeth and the cheek. The tube was fixed to the skin with tape. Tube side (left vs. right) in the oral cavity was alternated between subjects in each group. The infusion flow was individually adjusted to the subject's request (8-12 ml/min). By this, a swallowing frequency of 4-6/min was achieved. During the MEG measurements of 15 minutes duration, the subjects swallowed self-paced without external cueing. Swallowing acts were identified by surface electromyographic (EMG) recording with bipolar skin electrodes (Ag-AgCl) placed on the submental muscle groups [38,39]. The electrodes were connected to a bipolar amplifier (DSQ 2017E EOG/EMG system, CTF Systems Inc., Canada). EMG data was high pass filtered with 0.1 Hz. MEG data were collected using a whole head 275-channel SQUID sensor array (Omega 275, CTF Systems Inc., Canada) located in a magnetically shielded room. Magnetic fields were recorded with a sample frequency of 600 Hz, and data were filtered using a 150 Hz low-pass filter. Recordings were performed while subjects were seated in a comfortable upright position and watched silent movie of their choice. During MEG recordings subjects' and patients' head movements were recorded. Only measurements with a total head movement of less than 1 cm were taken into account for further calculations. No difference in the extent of head movement was observed between the groups. Furthermore, subjects were observed via an online video screen during recordings by a medical technician to exclude any other task-related movements.

### Data analysis

**Figure 1:**
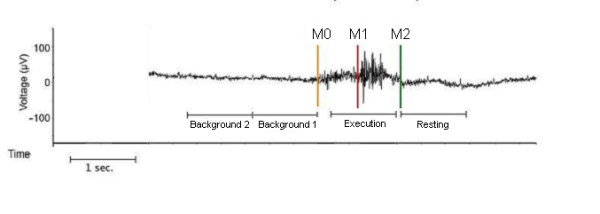
**Definition of active and resting stage of swallowing-related muscle activity**. The EMG recording of one swallowing act is shown (surface electrodes, recording from the submental muscles). For the analysis with SAM, the beginning (M_1_) and the end (M_2_) of larynx elevation were marked. The activation phase and the corresponding resting phase were defined. To estimate the maximal null distribution a third marker (M_0_) at the beginning of preparation activity was set and two background phases were defined (Methods).

Data analysis was performed as previously published [10,15,16]. Briefly, a band pass filter 3-100 Hz was applied to data. After that, each individual's EMG signal was used to mark the beginning (M_1; _> 100% increase of amplitude) and the end (M_2; _> 50% reduction of amplitude) of the swallowing-related muscle activation for every single swallow. A third marker (M_0_) was set to distinguish background activity from the onset of swallowing preparation. Background activity is the activation recorded by the surface EMG electrodes between the individual swallowing acts. Marker setting is the only step in data processing where variability can occur. Therefore, the person setting the markers was blinded for stroke localization and the presence of dysphagia. For analysis of the swallowing execution phase, time intervals were defined (see Figure 1):

(1) Execution stage: -0.4 to 0.6 s in reference to M_1_

(2) Resting stage: 0 to 1 s in reference to M_2_

(3) Background stage 1: -1 to -0 s in reference to M_0_

(4) Background stage 2: -2 to -1 s in reference to M_0_

About five percent of the trials were rejected due to overlap between (1) and (2) or between (4) and (2) of the subsequent swallow. The time intervals (3) and (4) were used to estimate the maximum null distribution.

According to previous SAM results on motor tasks, MEG data were filtered within the beta frequency range (13-30 Hz) and the two adjacent bands (alpha: 8-13 Hz; gamma: 30-60 Hz). From the filtered MEG data, SAM was used to generate 20 × 20 × 14 cm volumetric pseudo-t images [40], with 3-mm voxel resolution for all three frequency bands. A pseudo-t value cancels the common-mode brain activity by subtracting the source power found in a defined control stage from the source power in the active stage. To account for uncorrelated sensor noise, this difference is normalized by the mapped noise power [40,41]. For analyzing cortical activity during the execution stage (1), the corresponding resting stage (2) served as control.

The acquired MRI as part of routine stroke care did not contain the necessary localization information required for the normalization procedure. Therefore, normalization was performed as previously published by our group [42]. Briefly, the individual SAM images were mapped on the MNI (Montreal Neurological Institute) space using the knowledge about the fiducial point positions in the MEG coordinate system. The MNI space is also used in SPM2, and corresponds approximately to the Talairach space. Then, the rotated SAM image was shifted so that the center of the spherical head model used in the SAM calculation coincided with the center of the head model calculated for the template. In a last step, the size of the SAM image was adjusted so that the radii of the head models in both images matched. Compared to an established MRI based normalization procedure (SPM2), the new method shows only minor errors of about 0.5 cm in single subject results as well as in group analysis [42].

For SAM analysis of single conditions, the significance of activated brain regions was assessed by the permutation test method described by Chau and co-workers [43,44]. Therefore, it is necessary to determine the maximum null distribution. This distribution demonstrates cortical activation between the target movements. We therefore assessed two background stages before the onset of swallowing preparation as representing a control stage. This maximum null distribution was estimated by comparing Background stages 1 (active) and 2 (control). For the comparison of the different groups, a standard permutation test for unpaired samples was performed [44].

Hemispheric lateralization of swallowing related activation was quantified using a lateralization index (LI), which was calculated as (L-R)/(L+R), where L and R are the cumulative pseudo-t activation in the sensorimotor cortex (BA 3, 1, 2 and 4 according to the Talairach atlas) of the left and right hemispheres, respectively. A positive LI indicates left hemispheric lateralization, while a negative LI indicates stronger right hemispheric activation. A LI of 0 represents indeterminate dominance, 1 or -1 respectively are indicating unilateral activation [7,45].

## Results

### Clinical data

Clinical details and MRI findings are summarized in table 1. These data show that dysphagic patients with right hemispheric stroke were older than the patients of the other study groups, although this difference did not reach statistical significance (p = 0.053).

Fiberoptic endoscopic evaluation of swallowing (FEES) revealed signs of mild to moderate dysphagia in 19 of the 37 stroke patients. Salient endoscopic findings included difficulties in bolus preparation and food transport, spillage, delayed swallowing response, residues in the valleculae and pyriforme sinus, weakness of pharyngeal constriction and reduced laryngeal elevation. According to the 6-point scale of stroke-related dysphagia dysphagic stroke patients achieved scores between 2 and 4 representing mild to moderate dysphagia. All patients swallowed small amounts of water without penetration or aspiration. The Stroke Scale of the National Institute of Health (NIH-SS) score was similar between dysphagic and non dysphagic stroke patients with comparable locations of brain infarction. Only in left hemispheric stroke patients a difference was found with higher values for left hemispheric stroke with dysphagia (LHS+) compared to left hemispheric stroke without dysphagia (LHS-) (p < 0.05).

### Swallowing behaviour during MEG measurements

To swallow during MEG recordings was barely demanding for the subjects. The infusion rate was chosen to allow comfortable swallowing for both control subjects as well as dysphagic patients. Additionally, the time interval between two swallows could be controlled by the subjects. Therefore, all participants tolerated the MEG examination without any difficulties. No coughing and especially no signs of aspiration occurred during the measurements. EMG power (i.e. RMS [root mean square] of the amplitude across the time interval between M0 and M2), number of swallows, and duration per swallow were highly variant, but did not significantly differ between related groups (see table 2; EMG power: F(6, 37) = 1.76, p = .135; number of swallows: F(6, 37) = .501, p = .804; duration per swallow: F(6, 37) = .486, p = .815).

**Table 2:** The submental EMG was recorded during MEG measurements.

Group	No. of swallows	Duration per swallow	EMG Power
**Controls**	49.0 +/- 12.6	2.8 +/- 1.3	32.4 +/- 13.0

**LHS+**	40.5 +/- 19.4	3.7 +/- 2.9	51.3 +/- 21.1

**LHS-**	43.8 +/- 18.7	3.9 +/- 1.7	39.8 +/- 26.5

**RHS+**	45.7 +/- 20.3	4.4 +/- 2.3	52.0 +/- 30.1

**RHS-**	42.1 +/- 11.6	3.2 +/- 0.9	34.0 +/- 13.5

**BSS+**	45.8 +/- 16.4	3.8 +/- 1.5	63.1 +/- 19.1

**BSS-**	58.0 +/- 15.1	4.0 +/- 0.8	66.4 +/- 22.4

EMG power, number of swallows and the duration per swallow did not differ between the 7 groups.

### Results of MEG measurements

#### a) Control group

Individual synthetic aperture magnetometry (SAM) analysis of swallowing related activation in control subjects resulted in bilateral event related desynchronization (ERD) of pericentral areas in the alpha, beta, and gamma frequency ranges. Compared to activation in the alpha and gamma frequency bands broader and stronger activation was seen in the beta band in all subjects. In both hemispheres, the maximum of beta ERD was found mainly in Brodmann Area (BA) 4 (primary motor cortex) and 6 (supplementary motor cortex) (see table 3). One subject showed only weak activation peaking in the right dorsolateral prefrontal cortex (BA 9).

**Table 3:** Brodmann areas of the beta ERD and ERS in both hemispheres in control subjects and all 6 patient groups.

Subject	Beta ERD		Prefrontal beta ERS	
	
	left	right	left	right
Controls				

1	6	6	--	--

2	6	6	--	--

3	6	6	--	--

4	4	4	--	--

5	6	6	--	--

6	6	4	--	--

7	4	9	--	--

RHS +				

1	40	--	--	47

2	--	2	--	--

3	7	3	--	46

4	--	4	--	47

5	6	6	46	45

6	6	4	--	40

RHS-				

1	7	6	--	13

2	6	4	47	--

3	6	6	44	--

4	6	6	--	--

5	4	40	45	47

6	6	6	--	13

7	9	6	--	44

LHS +				

1	4	7	--	45

2	4	6	--	--

3	6	6	45	--

4	6	6	--	--

5	9	9	47	46

6	4	6	--	--

7	4	6	--	45

8	--	40	--	45

LHS-				

1	7	2	13	--

2	6	9	44	45

3	3	6	46	--

4	6	6	--	--

5	6	--	44	--

6	4	4	46	--

7	-	6	--	44

8	6	6	44	45

BSS+				

1	6	6	--	--

2	4	3	--	--

3	9	9	--	--

4	40	7	--	--

5	3	6	--	--

BSS-				

1	4	4	--	--

2	4	2	--	--

3	6	--	45	--

**Figure 2:**
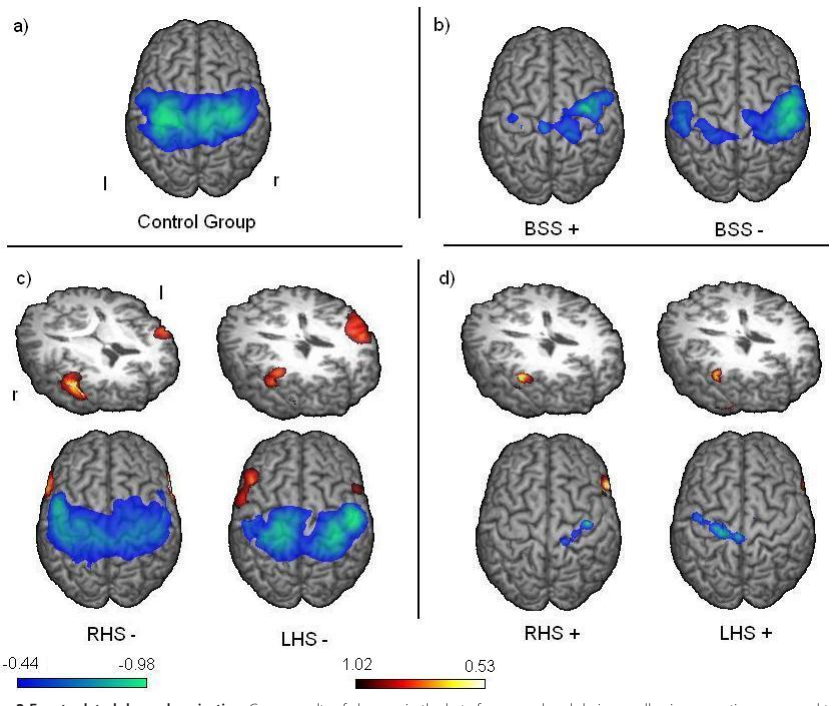
**Event related desynchronization**. Group results of changes in the beta-frequency-band during swallowing execution compared to the resting stage are shown. The color bar represents the t-value (yellow - red donates beta synchronization, green - blue the desynchronization). Significant activation in group analysis is shown (p < 0.05). a) In control subjects bilateral pericentral activation is seen. b) In both patient groups with brainstem stroke a right hemispheric lateralization of pericentral activation is observed. Stronger activation is seen in the non dysphagic group. c) In non dysphagic patients with hemispheric stroke pericentral activation is lateralized to the unaffected hemisphere. Additionally prefrontal synchronization with lateralization to the affected side is observable. d) In dysphagic hemispheric stroke patients very little pericentral activation of the affected hemisphere is found, while no contralesional activation is present. Prefrontal activation is only significant in the right hemisphere.

Group analysis showed significant bilateral beta ERD located in the primary and secondary sensorimotor areas including BA 1, 2, 3, 4, 5, 6 and 7 (p < 0.05) with a maximum of activation in BA 4 in the left hemisphere, and in BA 6 in the right one. Analysis of low gamma and alpha activation revealed similar but weaker activity of the sensorimotor cortices (p < 0.05). No significant brain activation, especially no event related synchronizations (ERS) were observed in other cortical areas (see Figure 2).

#### b) Hemispheric stroke without dysphagia

In all non-dysphagic patients with hemispheric stroke, cortical and subcortical, individual SAM analysis resulted in beta ERD of the pericentral areas in at least one hemisphere. All but one patient showed main activation in the primary and secondary sensorimotor areas. ERS of the prefrontal or insular cortices was seen in 13 of 15 non dysphagic hemispheric stroke patients, predominantly in the right hemisphere (see table 3). Additionally, pericentral alpha and gamma desynchronizations were found to be more inconsistent and weaker compared to beta desynchronization.

Group analysis of non-dysphagic patients revealed bilateral activation of the primary and secondary sensorimotor areas (BA 1, 2, 3, 4, 5, 6, and 7) (p < 0.05), with weak and not significant lateralization to the unaffected hemisphere, which is also reflected by the lateralization indices (right hemispheric stroke [RHS] -.2; left hemispheric stroke: .14). Activation in LHS- peaked in BA 6 in the right and BA 3 in the left hemisphere. In RHS-, maximum activation was found in BA 3 in the right and BA 6 in the left hemisphere. In both groups, distinct bilateral prefrontal beta synchronization was found (p < 0.05). In LHS-, maximum ERS was located in BA 45 in the right hemisphere and in BA 46 in the left hemisphere (see Figure 2).

#### c) Hemispheric stroke with dysphagia

In all dysphagic patients with hemispheric stroke, SAM analysis resulted in beta ERD of the pericentral areas in at least one hemisphere. Again most patients showed activation in the primary and secondary sensorimotor areas. 3 patients exhibited only weak and contralesional activations in tertiary areas like BA 9 (prefrontal cortex) and 40 (supramarginal gyrus). Pericentral alpha and gamma desynchronizations were found inconsistently and were weaker compared to beta desynchronization. ERS of at least one prefrontal or insular cortex was seen in 10 of 14 dysphagic hemispheric stroke patients (see table 3).

Group analysis revealed weak beta ERD located mainly in the primary motor cortex of the affected hemisphere (p < 0.05). Hardly any contralesional somatosensory ERD was found in both groups. In LHS+, activation was mainly located in the left BA 4 spreading marginally into BA 6. In right hemispheric stroke with dysphagia (RHS+) activation included BA 4 and 3 with strongest activation in BA 4. The strong lateralization in both patient groups is reflected by the lateralization indices (RHS: -0.98; LHS: 0.78). In both groups prefrontal ERS reached significance (p < 0.05) only in the right hemisphere. The maximum ERS was located in BA 46 in RHS+, and in BA 45 in LHS+ (see Figure 2).

#### d) Brainstem stroke (BSS)

With one exception (BSS+ subject no. 4) all patients with brainstem stroke demonstrated bilateral pericentral cortical beta ERD. Activations were mainly found in primary and secondary sensorimotor areas. Two patients with dysphagia exhibited with weak activation in tertiary areas (BA 9 and 40). Prefrontal activation was seen in one non-dysphagic patient with brainstem stroke (see table 3).

In group analysis of both dysphagic and non dysphagic patients with BSS significant bilateral beta ERD of pericentral areas (BA 1, 2, 3, 4, 5, and 6) was found. In both, dysphagic and non-dysphagic patients, activation peaked in BA 6 in the right hemisphere or BA 4 in the left one, respectively. Group SAM results of both groups were lateralized to the right sensorimotor cortex (LI: .75 in BSS+, respectively LI: .54 in BSS-) (see Figure 2). No significant brain activation, especially no ERS, was observed in other cortical areas in any of the examined frequency bands. Additionally, no significant ERD were found in the alpha and gamma frequency ranges in both groups of brainstem stroke patients.

## Discussion

In the present study, patients in the early subacute stage after stroke were measured by means of whole-head MEG. Our data revealed distinct patterns of cortical swallowing activation, which depended on the region affected by stroke. While hemispheric stroke in the early subacute phase associated with dysphagia resulted in decreased ipsilesional cortical activation and a nearly extinguished activation of the contralesional hemisphere, in contrast patients with hemispheric strokes without dysphagia were characterized by bilateral activation comparable to healthy controls. Additionally, all groups of patients with hemispheric stroke showed prefrontal activation, which was neither seen in healthy subjects nor in patients after brainstem stroke. In brainstem stroke a strong right hemispheric lateralization was found in both groups (with and without dysphagia).

### Localization of cortical activation

Cortical beta ERD in patients as well as control subjects were observed in the bilateral superior somatosensory areas. These activated cortical areas are located more superior and medial with respect to what has to be expected based on the homunculus. Additionally the swallowing related cortical activation is spread extensively in primary and secondary motor and sensory areas in both hemispheres, found in healthy controls and non-dysphagic patients with hemispheric stroke. This is a well-known phenomenon in functional brain imaging of human swallowing processing, which has been observed in several former MEG studies by our group [10,15-17,30,46]. But also other studies using TMS, PET and MEG demonstrated similar patterns of activation [8,12,47]. As an explanation for this widespread cortical activation in swallowing processing projections to and from the swallowing tract which is located in rostral non-primary motor areas, are suggested by Hamdy and co-workers [47]. The swallowing network therefore extends beyond the primary sensorimotor cortex and involves secondary areas.

### Hemispheric stroke with dysphagia

Irrespective of the affected hemisphere, a strongly reduced cortical activation was observed in dysphagic stroke patients. While little pericentral activation remained in the affected hemisphere, no significant activation of the primary sensory and motor areas was seen on the contralesional side.

The appearance of dysphagia after unilateral stroke irrespective of the affected hemisphere is a well-known phenomenon [2,14,48,49]. It is often seen after infarction of the middle cerebral artery. In the current experiment, the reduced activation of the affected hemisphere is, at least partly, explained by the reduction of grey matter due to the ischemic stroke, which makes less neurons and synapses available for the initiation and coordination of deglutition. More remarkable, though is the nearly abolished activation in the contralesional hemisphere found in patients after acute right and left hemispheric stroke. This could either be a sign of a general initial cortical shock after stroke [50], or point to a more network or function-specific reaction. To our knowledge to date no imaging studies on swallowing in patients in this acute stage after stroke have been performed. However, in the language domain, Saur and co-workers [51] examined aphasic patients and found a strongly reduced activation of the contralesional hemisphere when studying patients within the first two days after stroke. This pattern changed to an increased brain activation of the contralesional hemisphere in the subacute phase (mean 12.1 days after stroke), with subsequent normalization in the chronic phase [51]. Saur and co-workers concluded that the initial decrease of contralesional brain activation in the acute stage might be explained by disruption of the language network in terms of diaschisis. The concept of diaschisis was coined by von Monakow in 1914. In brief, it explains loss of function in undamaged brain areas connected to an acutely damaged area, as a consequence of a disruption of function in the remaining intact system [52,53]. We propose a comparable effect in the present study. We hypothesize that the distinct reduction of contralesional activation is caused by a disturbance of the swallowing network of the ipsilesional hemisphere, leading to a strongly reduced or even missing activation in these areas.

In contrast to the former study by Saur where patients were examined within two days, patients in the present study were measured about eight days after stroke. Unfortunately, no longitudinal data are available in the present study. Based on previous results [20,51,54], we hypothesize that in dysphagic stroke patients, following the acute underactivation of the contralesional hemisphere and a subsequent over-activation, a normalization of activation is predicted in the chronic stage in those patients who recover well.

### Hemispheric stroke without dysphagia

In patients without any signs of dysphagia after hemispheric stroke, extensive bilateral pericentral activation comparable to the group of healthy controls was observed in the early subacute phase after stroke. The observed lateralization to the contralesional hemisphere was only little pronounced and did not reach significance in the small group of examined patients. Still, these results are in line with previous TMS studies by Hamdy and co-workers. Patients that recovered from dysphagia presented with an increased motor representation of the pharynx in the unaffected hemisphere, while patients with persisting dysphagia showed no relevant changes of cortical motor representation [20]. Taken together, the Hamdy 1998 study and our own results suggest that activation of, and reorganization within the unaffected hemisphere is essential for enabling a normal swallowing function in patients after stroke.

### Premotor activation

In all groups of hemispheric stroke patients, beta synchronization of the dorsolateral prefrontal cortex (DLPFC; BA 44, 45 and 46) and/or insular cortex (BA 13) was found. Bilateral ERS was seen in both groups of non-dysphagic patients, while in dysphagic patients with hemispheric stroke only the right hemispheric premotor synchronization reached significance. Remarkably, this synchronization was neither seen in healthy control subjects nor in dysphagic and non-dysphagic brainstem stroke patients.

So far, the relevancy of beta ERS is not completely understood. On the one hand, it is known to be involved in the post-movement stage and higher cognitive functions [55-57]. On the other hand, it seems to play an important role in resting [58].

The insular cortex has connections to several brain regions linked to swallowing, including the premotor cortex, the frontal operculum, and secondary somatosensory and retroinsular area of the parietal lobe [59]. Regarding lateralization of insular activation, several studies observed either a bilateral activation, or a right hemispheric dominance [8,9,11,60,61]. In humans, damage to the frontal operculum as well as damage to the insula have been reported to cause dysphagia [62,63].

The DLPFC is known to play a crucial role in working memory [64]. An involvement of the prefrontal cortex in central swallowing processing has been seen in previous imaging studies on human swallowing [7]. Moreover, in primates stimulation of the frontal operculum is known to evoke swallowing [65].

It remains unresolved why ERS of the DLPFC and the insular was observed in hemispheric stroke patients in the present study. A former MEG study of our group utilizing an identical self-paced swallowing paradigm on a 151 MEG channel system found, significant ERS in the frontal operculum and insular cortex in healthy subjects during volitional swallowing [7]. In none of our previous studies on cortical swallowing processing using the 275 channel system, significant ERS in these areas were found [10,15,16,18]. The artifact caused by oropharyngeal muscle activation during deglutition makes it difficult to study activation in subcortical and bulbar structures [12,34]. We suggest that the higher number of channels of our MEG system might even aggravate the problem of the muscular artefact and by this reduce the observable subcortical activation. The DLPFC and insular activation in hemispheric stroke patients is therefore, supposedly, much stronger than that of healthy controls and patients with brainstem stroke. In hemispheric stroke patients, the swallowing-related pericentral areas of the affected hemisphere are destroyed by ischemic stroke. This leads to consecutive disruption of the cortical swallowing network, including a distinct reduction of sensorimotor activation in the contralesional hemisphere, as discussed above. We therefore conclude that the increase of DLPFC and insular activation observed in patients with hemispheric stroke is caused by compensational mechanisms in the acute post-stroke phase.

### Brainstem stroke

Both groups of patients with brainstem stroke were very small. Both groups demonstrated with a right hemispheric lateralization of sensorimotor cortical activation. The lateralization effect was stronger for the group of dysphagic patients. Overall, activation in non dysphagic brainstem stroke patients was stronger compared to dysphagic brainstem stroke patients, but reduced compared to the control group.

We propose that the observed effects in cortical swallowing processing after brainstem stroke can be explained by two different effects. First, the ischemic stroke of central pattern generators in bulbar areas has a strong impact on the cerebral network of swallowing processing. It results in an overall reduction of cortical activation in both patient groups, which reflects the concept of diaschisis, as already discussed above. Second, the right hemispheric lateralization found in both groups of brainstem stroke patients supports the hypothesis of hemispheric task sharing in swallowing processing [15,66]. The left hemisphere more selectively mediates the oral phase and therefore volitional components, whereas the right hemisphere contributes more to the pharyngeal phase and automatic reflexive aspects of swallowing. This was hypothesized by Daniels and co-workers after the utilization of a dual-task paradigm to examine deglutition [67], and also underlined by a previous MEG study of our group [15]. The influence of right hemispheric stroke on the pharyngeal transit duration and a prolonged oral transit time after left hemispheric stroke was already demonstrated about 13 years earlier by Robbins and co-workers [14].

The bulbar areas mainly coordinate the pharyngeal (reflexive) phase of deglutition [68]. Therefore, patients with brainstem stroke involving the central pattern generators show predominant impairment of the pharyngeal phase [69-72]. Thus, the relative preponderance of right-sided cortical activation in patients with brainstem-stroke may indeed be interpreted as cortical compensation of subcortically caused dysphagia. This effect is found in dysphagic as well as in non dysphagic patients, suggesting a fast and early onset of compensatory mechanisms. In case of non dysphagic patients, these adjustment mechanisms might even be sufficient to avoid clinically manifest dysphagia.

### Limitations

Apart from the technical restrictions, the small group sizes are a major limitation of the present study. Especially the group size in the brainstem stroke group was very small. 37 patients with ischemic stroke were recruited over 18 month. Due to the necessity to transport patients to the MEG laboratory, only stable patients that did not need continuously monitoring and were able to give informed consent could participate. Therefore, the patients in the present study are about 10 years younger compared to the mean age of stroke patients in general. Aging affects in cortical swallowing processing have been demonstrated for healthy subjects with a mean age of 24 compared to healthy subjects of about 72 years of age [16]. An increase of somatosensory cortical activation during swallowing execution in elderly subjects compared to the young control group was found. This effect was present in both hemispheres, pointing to adaptive cerebral changes in response to aging effects on the complex process of swallowing. The results also underline the relevance of age matched control groups in neuroimaging studies related to deglutition. The control group in the present study was therefore carefully age matched to the groups of stroke patients.

Also patients with severe aphasia had to be excluded because they were not able to give informed consent. This might have caused a bias, since patients with stroke in the language dominant hemisphere are under-represented. Due to the small group sizes a further division into subgroups (subcortical versus cortical hemispheric stroke and pontine versus non pontine brainstem stroke) was not possible. Further studies have to examine the distinct patterns of processing in these subgroups. Additionally, it would be interesting to examine further influence factors on the cortical swallowing processing, including the cerebral blood flow, white matter lesions and brain atrophy.

## Conclusion

In the best of our knowledge the present study is the first to examine the different patterns of cortical swallowing processing in the early subacute stage after stroke. The results extend our understanding of the physiology and pathophysiology of human deglutition. The observed compensational mechanisms may be relevant in future research on swallowing therapies. Additional studies should be performed to examine longitudinal data on swallowing rehabilitation in stroke patients after hemispheric and brainstem stroke. A subdivision of hemispheric stroke into cortical and subcortical infarct should be performed after the recruitment of a bigger group of patients.

## Competing interests

The authors declare that they have no competing interests.

## Authors' contributions

IT performed analysis and interpretation of data and drafted the manuscript. IT was funded by the Deutsche Forschungsgemeinschaft. SS made contributions to the design and performed data acquisition. TW has made contributions to conception and design. OS has contributed to analysis and interpretation of data and was involved in drafting the manuscript. MF made contributions to conception and performed data acquisition. EBR and CP revised the manuscript critically for important intellectual content. AF and RD made substantial contributions to conception and design, and have given final approval of the version to be published. All authors read and approved the final version of the manuscript.

## Pre-publication history

The pre-publication history for this paper can be accessed here:

http://www.biomedcentral.com/1471-2377/11/34/prepub
